# Supplementary material for: Spatial analysis of COVID-19 incidence and the sociodemographic context in Brazil
Source: PLoS One. 2021 Mar 1;16(3):e0247794. doi: 10.1371/journal.pone.0247794 (PMC7920392; doi:10.1371/journal.pone.0247794)
Supplement: Attachment — Submitted filename: Response To Reviewers.docx [file pone.0247794.s001.docx]

### Correspondence author

Carlos Eduardo Raymundo

Instituto de Estudos em Saúde Coletiva, Universidade Federal do Rio de Janeiro, Rio de Janeiro, State of Rio de Janeiro, Brazil.

E-mail: caducer@gmail.com

Dear Editor and reviewers,

Thank you very much for your comments, they were valuable to improve the paper. We included the suggested corrections in the manuscript. Below we justify our decisions about the comments and list the changes.

Please notice that the text was also modified to fulfill the requests of this journal editorial office.

**1. Please ensure that your manuscript meets PLOS ONE's style requirements, including those for file naming.**

**Answer:** Agreed. We updated all citations for PLOS ONE’s style requirements

**2. We note that Figures 1-8 in your submission contain map images which may be copyrighted.**

**Answer:** The authors themselves authored all figures. All data were extracted from a publicly source, available in the electronic database of the Brazilian Institute of Geography and Statistics (IBGE).

Reviewer #1: COMMENTS:

**1) In Methods section of the abstract, it would be fitting to mention the time period in which the data were collected in.**

**Answer:** Agreed. The text was modified:

Methods: “This is an analytical ecological study using data from various sources. The study period was February 25 to September 26, 2020”.

**2) In Results section I suggest to mention the motivation why only covariates listed in Table 1 were considered. I also suggest to use more uniform spacing in Table 1.**

**Answer:** Partially agree. The spacing in Table 1 has been corrected. The requested information about the motivation for the selection of variables was introduced in the Methods section and not in the Results section. The text was:

Methods: “The study used demographic, socioeconomic, and healthcare covariables to investigate possible associations with the outcome. These variables were selected due to their public availability and relevance as social determinants of health.”

**3) Position of the legend in Figure 2 and Figure 3 in Results section needs to be adjusted so its edges don’t disrupt the map.**

**Answer:** Agreed. The legend in Figure 2 and Figure 3 text was corrected

**4) Table 2 in Results section is exceeding the page size. In general, consider reporting values rounded to two decimals.**

**Answer:** Agreed. The decimal values have been rounded to two decimals as suggested

**5) I find the Discussion section a bit incoherent. For instance, on lines 269-270 the authors report the finding that lower numbers in proportional mortality ratio suggest worse levels of quality of life and economic development. On the following lines (270-278), studies are reported that suggest the contrary. I would suggest to place author’s finding into the context of studies with similar finding, in addition to the study by Baqui et al. that mentions the ethnicity effect (lines 283-286). I would also suggest to include some references for the paragraph on lines 279-282.**

**Answer:** Thank you, it makes much more sense. The studies cited in the paragraph in question also identified a higher incidence and mortality from COVID-19, as did the present study. We modified two paragraphs about proportional mortality ratio, including others references.

“Considering the covariable PMR, lower values for this indicator suggest worse levels of quality of life and economic development [28]. In the current study, proportional mortality ratio was inversely associated with COVID-19 incidence in most Brazilian municipalities (the higher the incidence, the lower the PMR), possibly because this covariable is a proxy for social development.

In the same direction, Marra et al. [29], found higher COVID-19 mortality in the North and Northeast regions of Brazil, related to higher prevalence of comorbidities and lower socioeconomic development in these regions. Andrade et al. [19] identified high-risk clusters in six municipalities in the central-south region of Sergipe state in Northeast Brazil, including the state capital Aracaju, which has the state’s highest population density and the lowest socioeconomic level. Maciel, Castro-Silva and Farias [11] found spatial dependence of COVID-19 incidence among municipalities of Ceará state in Northeast Brazil and moderate direct correlation with the municipal human development index.”

**6) I would suggest to elaborate more on the limitations of the study. For instance, what kind of bias can be introduced ‘by the limitations related to variations in the scope, types of testing and notification of cases between municipalities and over time’ (lines 309-311)?**

**Answer:** Agreed. To make it clearer, the sentences were modified to:

“The current study presents some limitations related to variations in the notification of cases between municipalities and over time, potentially introducing information bias due to underreporting and missing data, frequent limitation in ecological studies, especially in epidemic periods. However, the inclusion of possible confounders and the use of local models allowed the analysis and identification of relevant predictors at the population level.”

Reviewer #2: The manuscript aimed to analyze the spatial distribution of COVID-19 incidence in Brazil’s municipalities (counties) and investigate its association with sociodemographic determinants to better understand the social context and the epidemic’s spread in the country. The study presents the results of original research. Experiments, statistics, and other analyses are performed to a high technical standard and are described in sufficient detail. However, I understand that a minor revision needs to be carried out in order to add quality to the manuscript.

**Abstract:
The background does not represent the study proposal. In this section it may be interesting to better describe the spatial situation of COVID-19 in Brazil. Additionally, it is interesting to clarify what are the gaps to be filled by this investigation.**

**Answer:** Agreed. The text was modified:

“Background: Identified in December 2019 in the city of Wuhan, China, the outbreak of COVID-19 spread throughout the world and its impacts affect different populations differently, where countries with high levels of social and economic inequality such as Brazil gain prominence, for understanding of the vulnerability factors associated with the disease. Given this scenario, In the absence of a vaccine or safe and effective antiviral treatment for COVID-19, nonpharmacological measures are essential for prevention and control of the disease. However, many of these measures are not feasible for millions of individuals who live in territories with increased social vulnerability. the study aims to analyze the spatial distribution of COVID-19 incidence in Brazil’s municipalities (counties) and investigate its association with sociodemographic determinants to better understand the social context and the epidemic’s spread in the country.”

**Introduction:
It is important to describe the socio-demographic measures and the inequality measures used in the studies that supported this investigation. Additionally, it is essential to explain what are the knowledge gaps and how the present manuscript intends to contribute on this topic.**

**Answer:** Agreed. We added information and other references in this sentence:

“A study conducted in the Ceará state (Brazil), investigated the correlation of the human development index by municipalities with the incidence of COVID-19 [11]. A study carried out in the United States used the Gini index as a measure of income inequality [17]. Variables such as mean income, health care facilities, education level and race have also been included as potential risk factors for the disease [10,12–14,18,19]. Nevertheless, the association of these factors with COVID-19 remains to be better understood.”

**Methods:
It is essential to explain the reasons for including variables in the analysis model. I understand that the variables on availability of health professionals in the analyzed territories (doctors and nurses) are another way of estimating inequalities in Brazilian municipalities. However, this option is not sufficiently explained in the manuscript.**

**Answer:** Agreed. We included a sentence in this paragraph:

The study used demographic, socioeconomic, and healthcare covariables to investigate possible associations with the outcome. These variables were selected due to their public availability and relevance as social determinants of health. In the particular case of health determinants, it is important to highlight the relationship between variables of availability of health professionals (such as doctors and nurses) and socioeconomic development, since locations with greater development in general also have a more developed job markets, which, in turn, attracts such professionals. In this sense, the availability of these professionals is a proxy for social inequality [23].

**Discussion:
Describe how the Brazilian findings can be used for other research scenarios in the world.**

**Answer:** Agreed. This paragraph was included in the discussion:

“The findings of this research reinforced by the methodology employed helped in the global efforts to understand the spatial dynamics of COVID-19, in order to offer tools at a specific geographical level for targeted interventions.”
